# Enhancing Point Cloud Registration for Pipe Fittings: A Coarse-to-Fine Approach with DANIP Keypoint Detection and ICP Optimization

**DOI:** 10.3390/s25227012

**Published:** 2025-11-17

**Authors:** Zeyuan Liu, Xiaofeng Yue

**Affiliations:** School of Mechatronic Engineering, Changchun University of Technology, Changchun 130012, China; 1202201005@stu.ccut.edu.cn

**Keywords:** point cloud registration, iterative closest point, keypoint detection, pipe fittings

## Abstract

In 3D reconstruction, loss of depth data caused by highly reflective surfaces often undermines the accuracy of point cloud registration. Traditional registration methods suffer from reduced accuracy and computational efficiency under such conditions. This paper presents a novel coarse-to-fine point cloud registration approach that combines a density-aware keypoint detection method with iterative closest point optimization to enhance both precision and computational performance. The proposed keypoint detection method optimizes registration by progressively refining the initial pose estimate through multi-scale geometric feature detection. This process includes a density-aware mechanism for removing edge outliers and an adaptive threshold based on normal vector inner products. This improves both keypoint identification accuracy and matching efficiency, providing better initial registration for the iterative closest point algorithm in scenarios with significant data loss. The approach prevents the iterative closest point algorithm from converging to local optima, which improves both convergence speed and overall computational performance. Experimental results show that, under optimal conditions, the runtime is reduced by up to 78.01% across several datasets, including those from Stanford, Kinect, Queen, and ASL-LRD. Compared to other traditional methods, the proposed approach delivers higher registration accuracy, even for multi-view point clouds with severe data loss, which demonstrates its robustness and potential for engineering applications.

## 1. Introduction

Pipe fittings, such as elbows, are essential components of industrial pipeline systems and are extensively utilized in construction [1], manufacturing [2], and energy [3]. The geometric shapes and spatial configurations of these pipe fittings directly impact the precision of system installation, operational efficiency [4], and safety. Therefore, accurate reconstruction and measurement [5] of these components are of significant importance. However, this process presents significant technical challenges due to the geometric complexity and material properties of the fittings. In response, 3D point cloud registration algorithms have become essential tools for reconstructing the shape and spatial position of pipe components. These algorithms enable the accurate alignment of raw point cloud data acquired by sensors into a unified coordinate system, thereby facilitating precise measurement and digital modeling. However, the performance of depth cameras based on structured light is often limited by the reflective properties of highly reflective surfaces during point cloud acquisition. For instance, when the illuminated surface is flat or smooth, incident light may reflect unevenly or concentrate excessively, making it difficult for the camera’s sensor to properly capture the reflected rays. This issue is particularly pronounced on metal surfaces or other highly reflective materials, often leading to data loss and compromising the integrity of the point cloud [6]. To address this challenge, many studies have adopted High Dynamic Range (HDR) technology, which captures illumination across multiple exposures to mitigate the effects of uneven surface reflections. Although HDR techniques have shown some success in reducing data loss, the complexity of multiple exposures and post-processing still leads to deficiencies in certain regions of the point cloud, thereby imposing higher demands on point cloud registration algorithms.

Point cloud registration is a key technique for aligning data from multiple sources into a unified coordinate system. This ensures consistent geometric relationships between datasets. Currently, the most widely used point cloud registration method is the Iterative Closest Point (ICP) [7]. Due to its stability and applicability, ICP has been extensively employed in industrial inspection, robotic navigation, and 3D reconstruction. However, the ICP algorithm is sensitive to initial alignment and requires the point clouds to be relatively close in position. Additionally, it is susceptible to noise and outliers. The performance of the ICP algorithm can be significantly limited when dealing with depth data loss from highly reflective surfaces. To address these challenges, researchers have proposed numerous ICP variants [8,9,10]. However, most of these variants remain sensitive to both initial alignment and noise. A prevalent approach to mitigate these limitations is to perform coarse registration [11,12,13,14] using feature matching. This provides a better initial alignment for the ICP algorithm, which is then applied to achieve final registration.

Feature-based coarse registration typically involves steps like keypoint detection, feature description, feature matching, and robust pose estimation. For coarse registration methods, researchers have proposed approaches like Fast Point Feature Histograms (FPFH) [15], Histograms of Point Pair Features (HoPPF) [16], and Binary and Triangle Combined (BTC) Descriptor [17] to improve feature descriptions. They have also suggested methods such as Graph-Cut Random Sample Consensus (GC-RANSAC) [18], V-Random Sample Consensus (VSAC) [19], Quadratic-time Guaranteed Outlier Removal (QGORE) [20], and Streamlined Progressive Sample Consensus (SPROSAC) [14] to enhance robust pose estimation. However, since feature descriptions capture the local characteristics of keypoints and robust pose estimation depends on the correspondence of these keypoints, the accuracy of the initial values provided to the ICP algorithm is ultimately constrained by the quality of keypoint detection on the surface of incomplete data.

Point cloud keypoints refer to a set of points that reflect the fundamental geometric structure of a point cloud. They can be extracted based on defined detection criteria, with rotational and translational invariance. Keypoint detection methods aim to identify prominent points at specific scales. Zhong [21] proposed the Intrinsic Shape Signatures (ISS) algorithm. It measures the saliency of keypoints based on the eigenvalue decomposition of the scatter matrix derived from a point and its neighboring points. Keypoints are considered only if the ratio between two consecutive eigenvalues is below a specified threshold. Zaharescu and Boyer [22] introduced a 3D keypoint detection algorithm called MeshDOG, designed for uniform triangular meshes, which is invariant to rotation, translation, and scale transformations. Steder et al. [23] proposed a new method for detecting keypoints and calculating feature descriptors in 3D distance data, called the Normal Aligned Radial Feature (NARF). This method extracts distinctive keypoints in 3D point clouds. Sipiran and Bustos [24] developed a 3D keypoint detection algorithm called Harris 3D, using the Harris operator. This algorithm determines keypoints by computing the Harris response within the neighborhood of observation points. The Scale-Invariant Feature Transform (SIFT), originally designed for 2D images, was adapted by Rusu and Cousins [25] for 3D point clouds, resulting in the SIFT 3D algorithm. This adaptation replaces pixel intensity in the original algorithm with the principal curvature of points in the 3D point cloud. With the advancement of deep learning, researchers have begun exploring learning-based algorithms for 3D keypoint detection in point clouds. This has led to the development of methods such as KeypointNet [26], Unsupervised Stable Interest Point Detection (USIP) [27], Dense 3D Feature (D3Feat) [28], and Semantic Keypoint (SKP) [29]. However, the application of deep learning-based algorithms in industrial fields is limited by challenges related to interpretability, the need for large amounts of labeled data, computational resource constraints, and their robustness in handling noise. Although significant progress has been made in 3D keypoint detection, there is still room for improvement in areas like robustness, generalization, adaptive parameter adjustment, and handling data loss from reflective surfaces.

In this paper, we propose a coarse-to-fine point cloud registration method for industrial pipe fittings that integrates the newly proposed Density-Aware Normal Inner Product (DANIP) keypoint detection with the ICP algorithm. The main contributions of this research are summarized as follows:

1. An innovative 3D point cloud keypoint detection method, DANIP, is proposed, which combines a density-aware anomaly point removal mechanism with a multi-scale locally adaptive threshold detection based on normal vector inner products. This method demonstrates exceptional performance in keypoint detection accuracy, matching precision, and computational efficiency.
2. We introduce a coarse-to-fine point cloud registration method based on DANIP keypoint detection and the ICP algorithm. This method effectively addresses the limitations of the ICP algorithm, which is prone to local optima, while significantly improving convergence efficiency and computational performance in the registration process.
3. We conduct a registration study of common pipe fittings in real-world environments to evaluate the effectiveness of the coarse-to-fine point cloud registration method based on DANIP and ICP. The proposed method achieves higher registration accuracy than mainstream algorithms, even in multi-view scenarios with severe data loss.

The remainder of this paper is organized as follows. Section 2 presents the calculation method for the Density-Aware Normal Inner Product. Section 3 describes the proposed coarse-to-fine point cloud registration method based on DANIP and ICP. Section 4 discusses the experimental validation of the proposed method. Finally, Section 5 concludes the paper with a summary of the findings.

## 2. Density-Aware Normal Inner Product Keypoint Detection

After obtaining the pre-processed point cloud data, the Density-Aware Normal Inner Product Keypoint Detection (DANIP) method detects keypoints through the following steps. First, a density-based edge point removal mechanism is introduced, while the mean of the inner product of normal vectors within an adaptive neighborhood is used as the response value for local keypoint detection. Then, a non-maximum suppression technique, based on the distribution of normal vectors and the point cloud, is applied to further enhance the reliability of the detected keypoints.

### 2.1. Density-Aware Normal Inner Product

**Figure 1:**
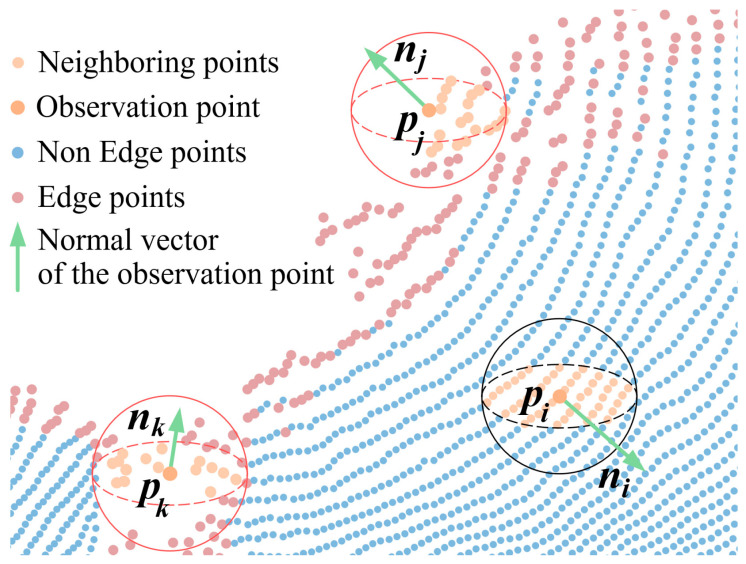
Distortion of edge point distribution.

In point cloud registration, the distribution of points located at the edges of the point cloud’s contours and in areas of missing data often deviates from the actual conditions due to limitations imposed by the sensor’s acquisition perspective and reflected light, as shown in Figure 1. Local features constructed based on this anomalous distribution, such as normal vectors, cannot accurately represent the true characteristics of these points. Therefore, we propose the Density-Aware Normal Inner Product keypoint detection method.

In keypoint detection, the neighborhood radius *r* is often manually set, which may require multiple adjustments for different point clouds. To overcome this limitation, we construct the detection response value of a point based on the number of nearest neighbors *k* in the point cloud. For a point ***p****_i_* in the point cloud ***P***, let the *k* nearest neighbors be denoted as ${\left\{p_{i}^{j}\right\}}_{j=1}^{k}$, with the distance between the neighbor ***p****_i_^j^* and ***p****_i_* being *d_i_^j^*. We define edge points based on density awareness as:

(1) $$\begin{array}{l} P={\left\{p_{i}\right\}}_{1}^{N}\\ \left\{\begin{array}{l} p_{i}\;\mathrm{is}\;\mathrm{an}\;\mathrm{edge}\;\mathrm{point},\;\mathrm{if}\;d_{i}^{k}>t_{i}\\ p_{i}\;\mathrm{is}\;\mathrm{nonedge}\;\mathrm{point},\;\mathrm{if}\;d_{i}^{k}\leq t_{i} \end{array}\right. \end{array}$$

where *d_i_^k^* represents the distance between ***p****_i_* and its neighboring point ***p****_i_^k^*, *t_i_* is the local dynamic threshold, which can dynamically reflect the density distribution of the surrounding area of ***p****_i_*.

(2) $$t_{i}=\mu_{i}+\alpha \cdot \sigma_{i}$$

(3) $$\begin{array}{l} \mu_{i}=\frac{1}{m}\sum_{j=1}^{m}d_{j}^{k}\\ \sigma_{i}=\sqrt{\frac{1}{m}\sum_{j=1}^{m}\left(d_{j}^{k}-\mu_{i}\right)} \end{array}$$

where *μ_i_* is the local mean, *σ_i_* is the local standard deviation, *α* is the tolerance parameter for noise, *m* is the size of the local neighborhood, and *d_j_^k^* represents the distance from the neighbor ***p****_i_^j^* of ***p****_i_* to its own *k*-th nearest neighbor ***p****_j_^k^*.

*k* is the number of neighboring points and can be adjusted based on the density of the point cloud. When the point cloud density is low, *k* can be set between 10 and 20. Conversely, for high-density point clouds or cases with significant missing data, noise, or edge anomalies, *k* can be set between 20 and 40. The value of *m* should ensure that the neighborhood covers local density variations while avoiding the inclusion of irrelevant regions. It is recommended to set m between 0.5*k* and *k*, rounded to an integer. The tolerance coefficient *α* can be adjusted according to the noise level. For low-noise scenarios, *α* can be set between 0.5 and 1, while for high-noise scenarios, *α* can be set between 1.5 and 2.

The core of density-aware edge point detection lies in comparing the distance *d_i_^k^* from point ***p****_i_* to its *k*-th nearest neighbor with a locally adaptive threshold *t_i_*. In regions with uniform density, *d_i_^k^* is close to the threshold *t_i_*, and the point is classified as a non-edge point. In contrast, at density-varying edge regions, *d_i_^k^* increases significantly due to the absence of neighboring points and exceeds the threshold *t_i_*, enabling precise detection. Additionally, dynamically adjusting the threshold *t_i_* using the neighborhood standard deviation *σ_i_* effectively suppresses noise interference and enhances the algorithm’s robustness.

For non-edge points, we use the inner product of local normal vectors as the response value for keypoint detection. Let the normal vector of ***p****_i_* be ***n****_i_*, and the normal vector of the neighbor ***p****_i_^j^* be ***n****_i_^j^*. We define the response value *R_i_* for Density-Aware Normal Inner Product as:

(4) $$R_{i}=\frac{1}{k}\sum_{j=1}^{k}\left|n_{i}\cdot n_{i}^{j}\right|$$

(5) $$n_{i}\cdot n_{i}^{j}=\left|n_{i}\right|\cdot \left|n_{i}^{j}\right|\cdot \cos\left(n_{i},\;n_{i}^{j}\right)$$

**Figure 2:**
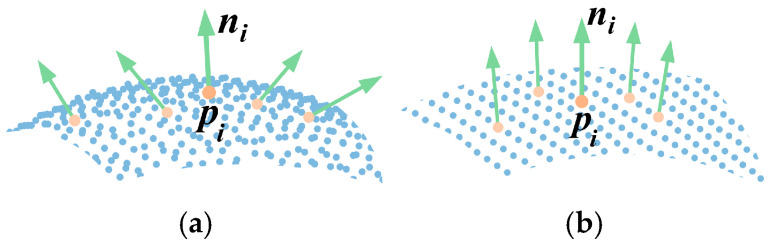
Normal vector diagram. (**a**) Curved Area; (**b**) Flat Area.

The value of *R_i_* ranges from [0, 1]. When ***p****_i_* is at a protruding or recessed position, the angle between ***n****_i_* and ***n****_i_^j^* becomes larger, resulting in a smaller value for *R_i_*, as shown in Figure 2a. Conversely, when ***p****_i_* is situated on a flatter surface, the angle between ***n****_i_* and ***n****_i_^j^* is smaller, leading to a larger value for *R_i_*, as illustrated in Figure 2b.

After obtaining the response value *R_i_* of the non-edge point ***p****_i_* in the point cloud, with its neighboring points denoted as ***p****_i_^j^* and their response values as *R_i_^j^*, the keypoint detection condition is defined as:

(6) $$t_{R_{i}}=\min\left(\frac{1}{m_{1}}\sum_{j=1}^{m_{1}}R_{i}^{j},\frac{1}{m_{2}}\sum_{j=1}^{m_{2}}R_{i}^{j},\frac{1}{m_{3}}\sum_{j=1}^{m_{3}}R_{i}^{j}\right)$$

(7) $$\left\{\begin{array}{l} p_{i}\;\mathrm{is}\;\mathrm{keypoint},\;\;\;\;\;\;\;\;\mathrm{if}\;R_{i}<t_{R_{i}}\\ p_{i}\;\mathrm{is}\;\mathrm{nonkeypoint},\;\mathrm{if}\;R_{i}\geq t_{R_{i}} \end{array}\right.$$

where *t_Ri_* is the local multi-scale detection threshold, and *m*_1_, *m*_2_, *m*_3_ represent the local scale sizes, with values of *k*/3, 2*k*/3, and *k*, respectively, rounded up to the nearest integer.

### 2.2. Non-Maximum Suppression

In order to enhance the accuracy and robustness of keypoint detection, this study also incorporates non-maximum suppression (NMS) to effectively eliminate redundant keypoints, thereby improving the precision of the detection results.

For a point ***p****_i_* in the point cloud ***P***, the nearest *k* neighboring points are ${\left\{p_{i}^{j}\right\}}_{j=1}^{k}$, with the distance between ***p****_i_* and ***p****_i_^j^* denoted as *d_i_^j^*. The covariance matrix for ***p****_i_* and its neighboring points ***p****_i_^j^* is established as in:

(8) $$\mathrm{cov}\left(p_{i}\right)=\frac{\sum_{j=1}^{k}W_{i}^{j}\cdot \left(p_{i}-p_{i}^{j}\right)\cdot {\left(p_{i}-p_{i}^{j}\right)}^{T}}{\sum_{j=1}^{k}W_{i}^{j}}$$

(9) $$W_{i}^{j}=\frac{1}{d_{i}^{j}}=\frac{1}{\left\|p_{i}-p_{i}^{j}\right\|}$$

where *W_i_^j^* represents the weight. Calculate all the eigenvalues $\left\{\lambda_{i}^{1},\lambda_{i}^{2},\lambda_{i}^{3}\right\}$ ($\lambda_{i}^{1}\geq \lambda_{i}^{2}\geq \lambda_{i}^{3}$) of matrix cov(***p****_i_*). The magnitude of these eigenvalues reflects the variance of the data in the corresponding feature vector directions. Larger eigenvalues indicate that the point cloud is more widely distributed in that direction. Therefore, we define the response value *S_i_* for point ***p****_i_* based on non-maximum suppression as follows:

(10) $$S_{i}=\left\{\begin{array}{l} 1,\;\mathrm{if}\;d_{i}^{k}>t_{i}\\ s_{i},\;\mathrm{if}\;d_{i}^{k}\leq t_{i} \end{array}\right.$$

(11) $$s_{i}=\frac{\lambda_{i}^{1}}{\lambda_{i}^{2}}$$

where *d_i_^k^* represents the distance between ***p****_i_* and the neighboring point ***p****_i_^k^*, and *t_i_* denotes the local dynamic threshold determined by Equation (2). *s_i_* reflects the degree of variation of the local point cloud around ***p****_i_* in the main direction. A larger *s_i_* indicates a more pronounced variation in the main direction around ***p****_i_*, suggesting that the local point cloud is more likely to exhibit linear or elongated characteristics, making ***p****_i_* more likely to be detected as a keypoint. Unlike method [21], which employs *s_i_* as the output response for keypoint detection, we innovatively redefine it as a modulation parameter in the NMS process, allowing *s_i_* to adaptively suppress spurious keypoints. Therefore, for any keypoint ***p****_i_*, the NMS response value *S_i_* and the NMS response values ${\left\{S_{i}^{j}\right\}}_{j=1}^{k}$ for neigh boring points ${\left\{p_{i}^{j}\right\}}_{j=1}^{k}$ are defined. We define the condition based on NMS as:

(12) $$\left\{\begin{array}{l} p_{i}\;\mathrm{is}\;\mathrm{keypoint},\;\;\;\;\;\;\;\;\mathrm{if}\;S_{i}=\max{\left\{S_{i},S_{i}^{j}\right\}}_{j=1}^{k}\\ p_{i}\;\mathrm{is}\;\mathrm{nonkeypoint},\;\mathrm{if}\;S_{i}\neq \max{\left\{S_{i},S_{i}^{j}\right\}}_{j=1}^{k} \end{array}\right.$$

where *k* represents the number of neighbors of point ***p****_i_*. Applying non-maximum suppression to all keypoints can determine the final keypoints.

The pseudocode of DANIP algorithm is shown in the Algorithm 1 below.
**Algorithm 1.** DANIP AlgorithmInput: point cloud ***P*** and number of neighboring points *k* Output: keypoint ***K_P_***1Calculate the local dynamic threshold *t_i_* based on Equation (3);2Use the local dynamic threshold *t_i_* to filter out edge outliers according to Equation (1);3Compute the normal vector *n**_P_*** of the point cloud ***P***;4Calculate the response value *t_Ri_* for dynamic multi-scale keypoint detection based on Equations (4)–(6);5For non-edge points, determine the candidate keypoints using Equation (7);6Construct the local neighborhood covariance matrix using Equations (8) and (9);7Determine the threshold for non-maximum suppression based on Equations (10) and (11);8Perform non-maximum suppression on the candidate keypoints based on Equation (11) to obtain the final keypoints ***K_P_***.

Compared to existing keypoint detection methods, DANIP first introduces an edge point removal mechanism, effectively eliminating the interference of edge outliers in the keypoint detection results. Secondly, in terms of detection range, DANIP adopts a dynamic multi-scale neighborhood strategy, selecting keypoints only when a point shows significant prominence across multiple scales. Finally, DANIP further refines the selection of keypoints with significant response values through local maximum suppression within the neighborhood range.

## 3. Coarse-to-Fine Registration Using DANIP Keypoints and ICP

**Figure 3:**
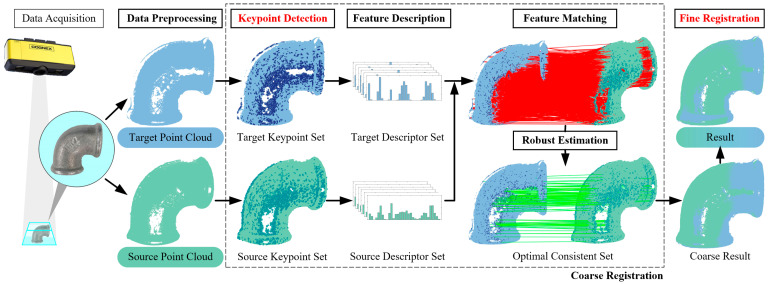
Coarse to fine registration.

Point cloud registration is a critical task in computer vision and 3D reconstruction, aiming to align point cloud data from different viewpoints or time instances to generate a unified 3D model. This section introduces a novel point cloud registration algorithm based on DANIP keypoints and the ICP method. The proposed algorithm follows a coarse-to-fine strategy, initially achieving a coarse alignment through efficient keypoint detection and matching, and then refining the alignment using ICP. This design not only prevents ICP from converging to a local optimum but also accelerates the registration process while enhancing the algorithm’s robustness to noise and outliers. A flowchart of the algorithm is shown in Figure 3, which includes several key steps: data preprocessing, keypoint detection, feature description, feature matching, robust estimation, and fine registration.

### 3.1. Coarse Registration Based on DANIP Keypoints

#### 3.1.1. Data Preprocessing

Due to the influence of environmental factors, the 3D point cloud data obtained by sensors during the acquisition process often contains redundant information. Therefore, data preprocessing is usually required before point cloud registration. Preprocessing typically includes filtering and downsampling. The purpose of filtering is to remove noise and outliers, while downsampling aims to reduce the data size and improve the computational efficiency of subsequent processing. In our method, we utilize voxel filtering for downsampling, a widely used technique.

#### 3.1.2. Keypoint Detection

Keypoint detection is a crucial step in feature-based coarse registration methods and plays a vital role in determining the accuracy of the coarse registration. In this paper, we use the DANIP method, as introduced in Section 2, to detect keypoints in the pipe fitting point cloud.

#### 3.1.3. Feature Description

Three-dimensional point cloud feature descriptors are used to extract and represent the local geometric features within the point cloud, enabling effective comparison and matching of keypoints in point cloud registration tasks. In this study, we employ the commonly used local feature descriptor FPFH [15] to represent the local features of the pipe fitting point cloud.

**Figure 4:**
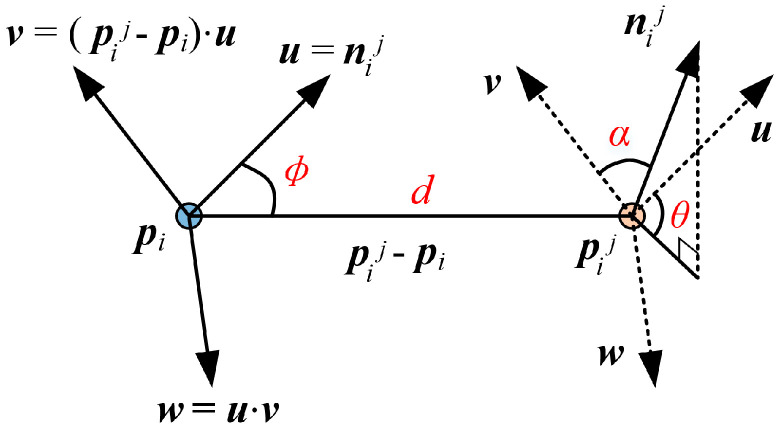
Definition of Local Coordinate System.

The core idea of the FPFH descriptor is to utilize a local coordinate system to describe the local features of a point. The definition of the local coordinate system is shown in Figure 4. Let ***p****_i_* be the detected keypoint, and ***p****_i_^j^* be the *j*-th neighboring point of ***p****_i_*. ***n****_i_* and ***n****_i_^j^* represent the normal vectors of ***p****_i_* and ***p****_i_^j^*, respectively. The local coordinate system is defined as follows:

(13) $$\left\{\begin{array}{l} u=n_{i}\\ v=\left(p_{i}^{j}-\;p_{i}\right)\cdot u\\ w=u\cdot v \end{array}\right.$$

Based on the defined local coordinate system, the FPFH descriptor employs the following parameters to represent the positional information between ***p****_i_* and ***p****_i_^j^*.

(14) $$\left\{\begin{array}{l} \alpha =v\cdot n_{i}^{j}\\ \theta =arctan\left(w\cdot n_{i}^{j},u\cdot n_{i}^{j}\right)\\ \phi =u\cdot \left(p_{i}^{j}-p_{i}\right)/\left\|p_{i}^{j}-p_{i}\right\| \end{array}\right.$$

**Figure 5:**
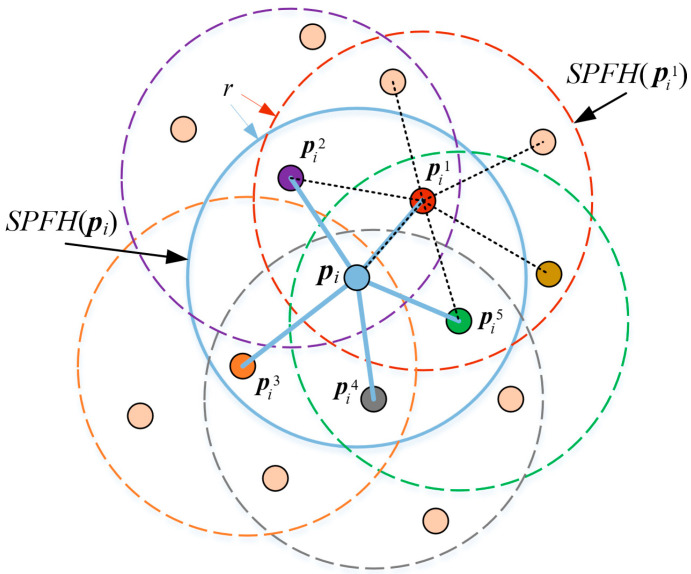
Calculation Range of *FPFH*(***p****_i_*).

The values of (*α*, *θ*, *ø*) for all neighboring points within a radius *r* around point ***p****_i_* are computed. A histogram with 11 bins is generated for all (*α*, *θ*, *ø*), and the statistical result is denoted as *SPFH*(***p****_i_*). Therefore, *FPFH*(***p****_i_*) is defined as:

(15) $$\begin{array}{l} FPFH\left(p_{i}\right)=SPFH(p_{i})+\frac{1}{k}\sum_{j=1}^{k}\frac{1}{\omega_{j}}\cdot SPFH(p_{i}^{j})\\ w_{j}=\left\|p_{i}^{j}-p_{i}\right\| \end{array}$$

where *k* is the number of neighboring points of ***p****_i_*. Figure 5 shows the calculation range of *FPFH*(***p****_i_*).

#### 3.1.4. Feature Matching

Feature matching is employed to establish correspondences between similar keypoints across different point cloud datasets.

Given the source point cloud ***Q***, the target point cloud ***P***, and their corresponding keypoint sets ***K_Q_*** and ***K_P_***, the features of keypoints ***q****_j_* and ***p****_i_* are represented by *FPFH*(***q****_j_*) and *FPFH*(***p****_i_*), respectively. In the feature space, if the distance between *FPFH*(***p****_i_*) and *FPFH*(***q****_j_*) is minimal, then ***p****_i_* and ***q****_j_* are considered a matching point pair, denoted as ***c****_i_*.

(16) $$\begin{array}{l} K_{Q}={\left\{q_{j}\right\}}_{1}^{M}\\ K_{P}={\left\{p_{i}\right\}}_{1}^{N}\\ c_{i}=(p_{i},q_{j}) \end{array}$$

In ${\left\{\mathrm{FPFH}\left(q_{j}\right)\right\}}_{1}^{M}$, the nearest neighbor for each ${\left\{\mathrm{FPFH}\left(p_{i}\right)\right\}}_{1}^{N}$ is identified, thereby establishing the correspondence set ***C*** between the point clouds.

(17) $$C={\left\{c_{i}\right\}}_{1}^{N}$$

#### 3.1.5. Robust Estimation

Robust estimation aims to accurately estimate the pose parameters of a target in the presence of noise, outliers, or other disturbances. In this paper, Maximum Likelihood Estimation Sample Consensus (MLESAC) [30] is employed to estimate the rotation and translation parameters for coarse registration. The objective of MLESAC is to maximize the likelihood function, and the objective function can be expressed as:

(18) $$\begin{array}{l} T^{*}=\arg\underset{T}{\max}\sum_{i=1}^{N}\log\left(P_{I}+P_{O}\right)\\ P_{I}=\varepsilon \cdot \frac{1}{\sqrt{2\pi }\sigma }\exp\left(-\frac{{\left\|p_{i}-T\cdot q_{j}\right\|}^{2}}{2\sigma^{2}}\right)\\ P_{O}=\left(1-\varepsilon \right)\cdot \frac{1}{\nu } \end{array}$$

where ***T*** represents the model parameters, *ε* is the inlier confidence rate, ***p****_i_* and ***q****_j_* are a pair of corresponding keypoints, *σ* is the standard deviation of the noise, and *ν* denotes the constant representing the distribution range of outliers.

### 3.2. Fine Registration

The ICP algorithm is a widely used method for point cloud registration. The ICP algorithm computes the rotation and translation parameters ***T*** by minimizing the distance between the registered point clouds ***P*** and ***Q***.

(19) $$\begin{array}{l} P={\left\{p_{i}\right\}}_{1}^{N}\\ Q={\left\{q_{j}\right\}}_{1}^{M} \end{array}$$

The objective function for the ICP algorithm is defined as follows:

(20) $$\underset{T}{\min}\left(\sum_{i=1}^{N}\left\|T\cdot q_{j}-p_{i}\right\|\right)$$

where ***p****_i_* represents the point on the point cloud ***Q*** that is closest to the transformed point ***q****_j_* after applying the rotation and translation. The method presented in this paper first uses DANIP keypoints to establish the correspondence set and estimate the rotation and translation parameters ***T***. It then resolves the problems of local optimality and low efficiency in the ICP algorithm by providing a good initial estimate of the transformation parameters ***T***.

## 4. Experimental Evaluation

In this section, we systematically evaluate the performance of the proposed DANIP method across multiple aspects. First, we conduct a comprehensive performance comparison of DANIP with existing mainstream keypoint detection algorithms, including SIFT 3D [25], Harris 3D [24], ISS [21], and NVDP [12], using four publicly available datasets. We then investigate the impact of different keypoint detection algorithms on the accuracy of coarse point cloud registration. Building on this, we focus on evaluating the improvements in the performance of the traditional ICP algorithm by integrating DANIP with a coarse-to-fine registration strategy. Finally, to verify the effectiveness of the proposed method in practical industrial applications, we use real pipe fitting point cloud data acquired by a structured light camera to test and analyze the registration performance of the DANIP-ICP combined coarse-to-fine registration algorithm under data missing conditions.

### 4.1. Keypoint Detection Performance

**Figure 6:**
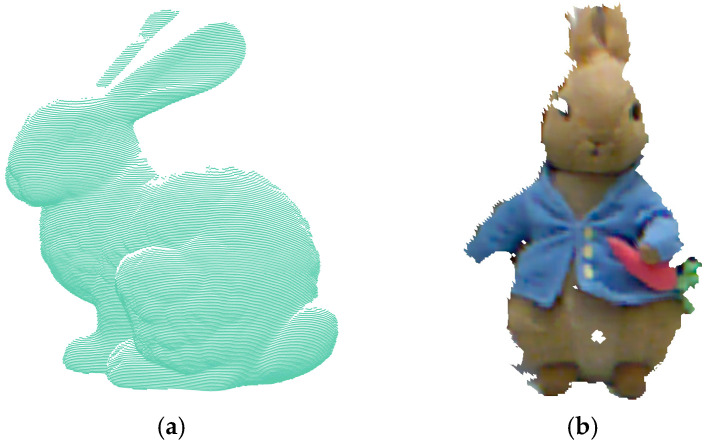

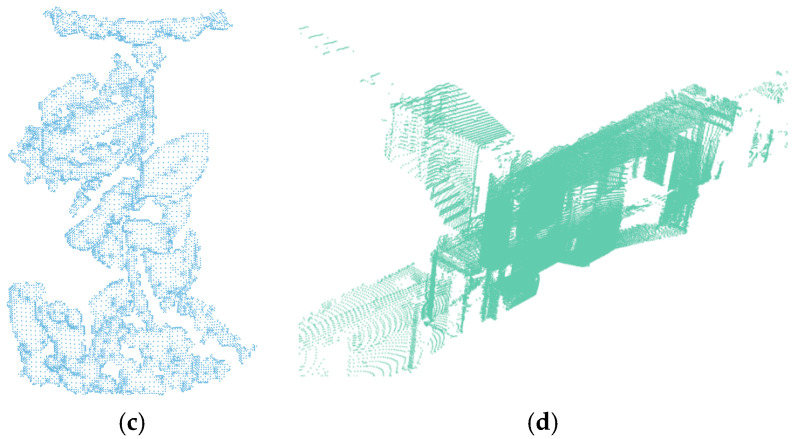
Visualization of some point clouds involved in the experiment.(**a**) bun045; (**b**) PeterRabbit000; (**c**) im0; (**d**) Hokuyo_1.

To evaluate the performance of the DANIP algorithm compared to existing mainstream keypoint detection methods, we conducted comparative experiments on four representative publicly available datasets: Stanford [31], Kinect [32], Queen [33], and ASL-LRD [34]. Table 1 provides a detailed description of the specific characteristics of these datasets. These datasets, collected using different types of sensors, exhibit significant differences in resolution, noise levels, and data sparsity, enabling an effective validation of the algorithm’s adaptability and robustness under varying scene conditions. Figure 6 presents some of the point cloud samples used in the experiment, visually highlighting the diversity of the datasets.

**Figure 7:**
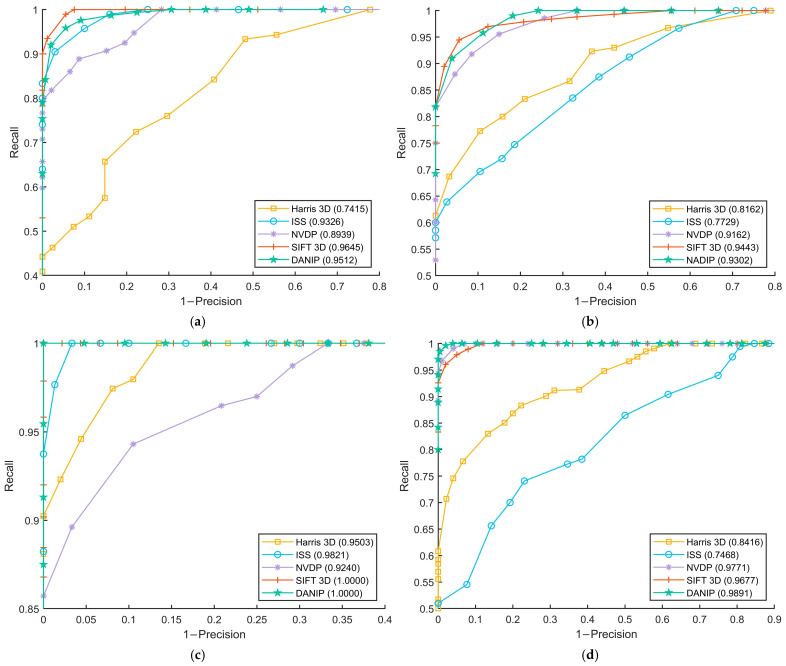
The RPC results for keypoint detection. (**a**) Stanford bun000&045; (**b**) Kinect PeterRabbit000&001; (**c**) Queen im0&2;(**d**) ASL-LRD stairs Hokuyo_0&1 (1/4 downsample).

For evaluation metrics, we used *Recall*, *Precision*, *Time*, and *F*_1_ score to assess the performance. For a keypoint ***q****_j_* on the source point cloud ***Q*** and a keypoint ***p****_i_* on the target point cloud ***P***, the criteria for ***p****_i_* and ***q****_j_* to be considered a correctly matched keypoint pair are:

(21) $$\left\|T^{\prime }\cdot q_{j}-p_{i}\right\|\leq t$$

(22) $$Recall=\frac{\mathrm{correctly}\;\mathrm{matched}\;\mathrm{keypoints}\;\mathrm{count}}{\mathrm{corresponding}\;\mathrm{keypoints}\;\mathrm{count}}$$

(23) $$Precision=\frac{\mathrm{correctly}\;\mathrm{matched}\;\mathrm{keypoints}\;\mathrm{count}}{\mathrm{total}\;\mathrm{number}\;\mathrm{of}\;\mathrm{matched}\;\mathrm{keypoints}}$$

where $T^{\prime }$ represents the true registration transformation parameters. *t* is the distance threshold. The value of *t* is varied to obtain the curves. The experimental results are shown in Figure 7 and the corresponding *F*_1_ and time are presented in Table 2.

As illustrated in Figure 7a,b, the performance of DANIP on the Stanford and Kinect datasets is second only to the SIFT 3D method. Notably, the results in Figure 7c,d demonstrate that DANIP achieves optimal performance on the Queen and ASL-LRD datasets. Quantitative analysis in Table 2 further confirms that DANIP attains higher *F*_1_ scores on most datasets, particularly excelling on the ASL-LRD dataset, which suffers from severe data missing issues. Moreover, compared to SIFT 3D, DANIP exhibits significant advantages in time efficiency, indicating that the method achieves a well-balanced trade-off between computational complexity and detection accuracy.

The experimental results reveal that DANIP demonstrates excellent performance in 3D keypoint detection tasks. Although its time efficiency is slightly lower than that of ISS and Harris 3D on some datasets, DANIP shows clear advantages in *F*_1_ scores on most datasets, particularly exhibiting stronger robustness when processing low-resolution or noisy data. This characteristic endows DANIP with significant practical value in real-world applications, especially in scenarios that require a balance between computational efficiency and detection accuracy.

### 4.2. Coarse Registration Comparison

To evaluate the performance of the proposed keypoint detection algorithm, DANIP, in the coarse registration task, we compared the coarse registration method based on the DANIP algorithm with several other commonly used algorithms, including ISS, Harris 3D, SIFT 3D, SUSAN [35], and NVDP, across different datasets. To quantitatively assess registration accuracy, root mean square error *RMSE*, mean reprojection error *MRE*, Bayesian information criterion *BIC* and time consumption *Time* were used as evaluation metrics.

(24) $$\mathrm{RMSE}=\sqrt{\frac{\sum_{1}^{N}\left\|T\cdot q_{j}-p_{i}\right\|}{N}}$$

(25) $$\mathrm{MRE}=\frac{1}{N}\sum_{1}^{N}\left\|T\cdot q_{j}-p_{i}\right\|$$

(26) BIC=kln(N)−2ln(L)

where ***T*** represents the transformation parameters estimated during coarse registration, *N* is the number of points in the source point cloud, ***q****_j_* is an arbitrary point in the source point cloud, and ***p****_i_* is the closest point in the target point cloud to the transformed ***q****_j_*. *k* and *L* are the number of parameters and maximum likelihood value of the point cloud transformation model, respectively. 

Considering the uncertainty of a single registration experiment, we conducted 100 trials to ensure the reliability of the results. Table 3 presents the experimental results, listing the average values of each evaluation metric.

As illustrated in Table 3, from the perspective of root mean square error *RMSE* and mean reprojection error *MRE*, the DANIP algorithm demonstrates superior performance across all datasets. Specifically, on the Stanford dataset, DANIP achieves an *RMSE* of 0.0022 and an *MRE* of 0.000855, slightly outperforming other algorithms. On the Kinect and Queen datasets, DANIP performs comparably to SIFT 3D, with RMSE values of 0.0047 and 0.0181, respectively, both of which are the lowest recorded. Notably, on the ASL-LRD dataset, DANIP achieves an *RMSE* of 0.1288 and an *MRE* of 0.065782, significantly surpassing other algorithms, which indicates that DANIP possesses higher registration accuracy when dealing with complex scenes. The Bayesian Information Criterion *BIC* also demonstrates the efficiency of the DANIP algorithm across all datasets. In comparison to other algorithms, DANIP shows superior BIC values on each dataset, further validating its stability and accuracy in point cloud registration tasks.

Furthermore, in terms of computational efficiency, the DANIP algorithm exhibits superior performance across all datasets. For instance, on the Stanford dataset, DANIP’s computational time is 2.44 s, which is markedly lower than the 4.79 s of SIFT 3D and the 4.32 s of Harris 3D. Similarly, on the Kinect and Queen datasets, DANIP’s computational times are 1.26 s and 1.21 s, respectively, again significantly lower than those of other algorithms. This suggests that DANIP not only excels in registration accuracy but also demonstrates remarkable computational efficiency.

**Figure 8:**
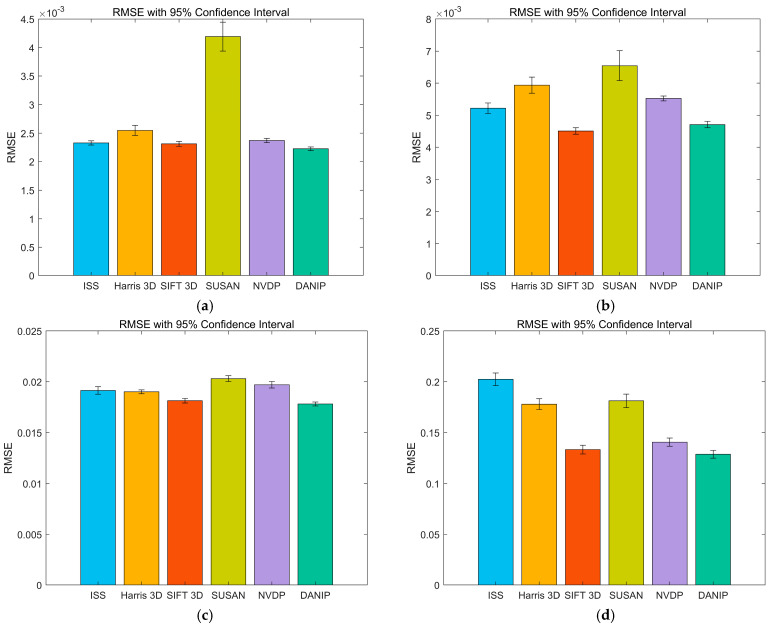
The 95% confidence interval bar chart.(**a**) Stanford; (**b**) Kinect; (**c**) Queen; (**d**) ASL-LRD.

To provide a more intuitive comparison of the coarse registration performance across different datasets, we have summarized the *RMSE* of 100 registration results in Table 4 and plotted the 95% confidence interval bar chart, as shown in Figure 8.

In Table 4, the stability and superiority of the DANIP algorithm across all datasets can be intuitively observed. This indicates that it maintains high accuracy when handling complex data. The confidence intervals in Figure 8 also clearly reflect the stability of DANIP, with the error ranges being small across all datasets, further validating its effectiveness in point cloud registration tasks.

The experimental results indicate that the DANIP algorithm exhibits significant advantages in both registration accuracy and computational efficiency, particularly when handling complex scenes and large-scale data, where its performance is notably superior to that of other algorithms.

### 4.3. Coarse-to-Fine Registration Comparison

**Figure 9:**
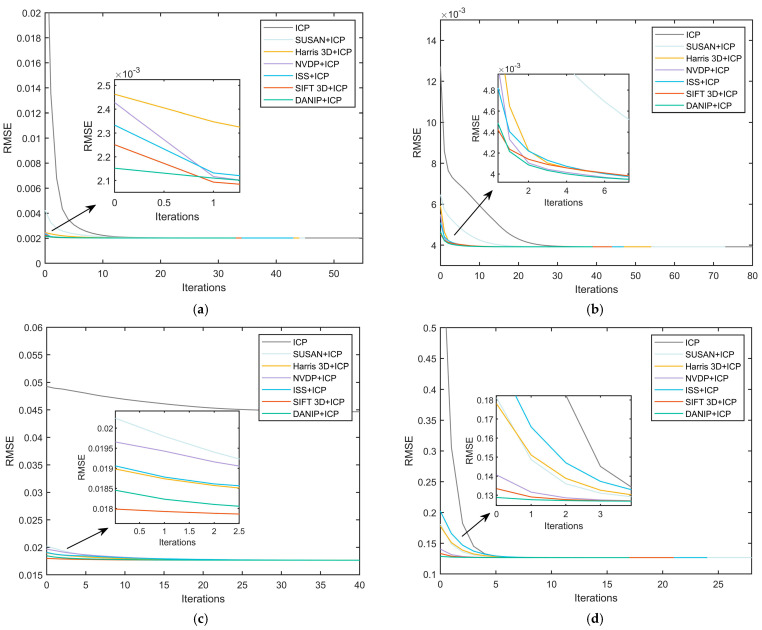
Coarse-to-Fine Registration.(**a**) Stanford bun000&045; (**b**) Kinect PeterRabbit000&001; (**c**) Queen im0&2; (**d**) ASL-LRD stairs Hokuyo_0&1 (1/4 downsample).

To validate the effectiveness of the proposed coarse-to-fine point cloud registration method based on DANIP and ICP, this study conducted experiments on four publicly available datasets and performed a systematic comparison with six common registration methods, including the traditional ICP algorithm, SIFT 3D + ICP, and Harris 3D + ICP. To comprehensively evaluate the performance of each algorithm, we used root mean square error *RMSE* and runtime as the primary evaluation metrics. The experimental results are presented in Figure 9 and Table 5.

The experimental results demonstrate the effectiveness of the coarse-to-fine point cloud registration algorithm based on DANIP and ICP. By utilizing the more accurate keypoint detection algorithm DANIP, the proposed method provides superior initial values for the ICP algorithm, thereby addressing the inefficiency and high sensitivity to initial values inherent in traditional ICP. As shown in Figure 8, the registration error *RMSE* for all methods tends to converge as the number of iterations increases. Notably, the DANIP + ICP algorithm exhibits the fastest convergence rate in Figure 8a,b,d. In Figure 8c, when the traditional ICP algorithm converges to a local minimum, the DANIP+ICP-based algorithm’s convergence error is significantly smaller than that of ICP.

Table 5 presents a comparison of runtime performance across different datasets. The DANIP+ICP algorithm achieves the shortest runtime among all evaluated methods. Specifically, compared to the traditional ICP algorithm, the proposed method reduces the runtime by 66.93%, 78.01%, 75.48%, and 23.69% on datasets A, B, C, and D, respectively. These results indicate that the coarse-to-fine registration algorithm based on DANIP+ICP not only accelerates convergence but also effectively avoids local minima, significantly improving computational efficiency.

The superior performance of the proposed algorithm can be attributed to the robustness of the DANIP algorithm in handling incomplete data, providing more reliable keypoints for initial alignment. Furthermore, DANIP generates more precise initial values, which contribute to faster and more accurate convergence during subsequent ICP refinement. Finally, the complementarity between the coarse and fine registration stages ensures global consistency and accuracy in the final registration result. The experimental results suggest that combining the advanced keypoint detection algorithm DANIP with the traditional ICP framework can greatly enhance point cloud registration performance, particularly in challenging scenarios involving incomplete or noisy data.

### 4.4. Pipe Fitting Registration Performance

To evaluate the effectiveness of the proposed coarse-to-fine point cloud registration method based on DANIPI and ICP in practical industrial scenarios, we compared it with several mainstream registration algorithms, including ICP, NDT [36], LM-ICP [37], G-ICP, and P-ICP [38]. To objectively assess registration accuracy, the root mean square error *RMSE* was used as the quantitative evaluation metric.

**Figure 10:**
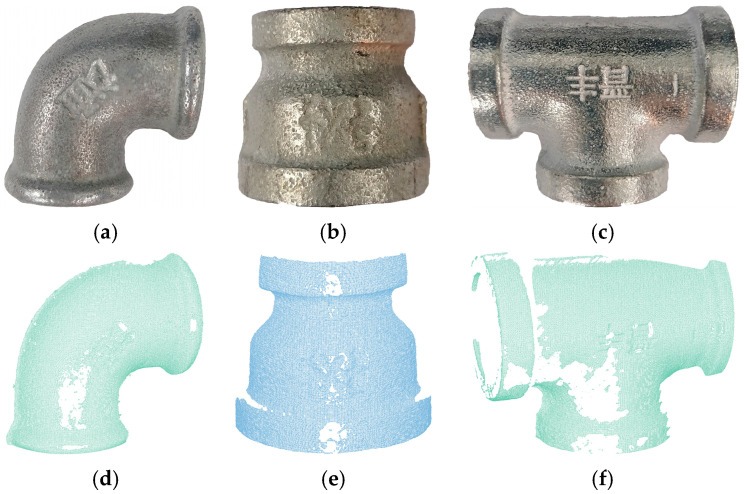
Common pipe fittings and point clouds. (**a**) Elbow; (**b**) Reducer; (**c**) Tee; (**d**) elbow02; (**e**) reducer01; (**f**) tee02.

**Figure 11:**
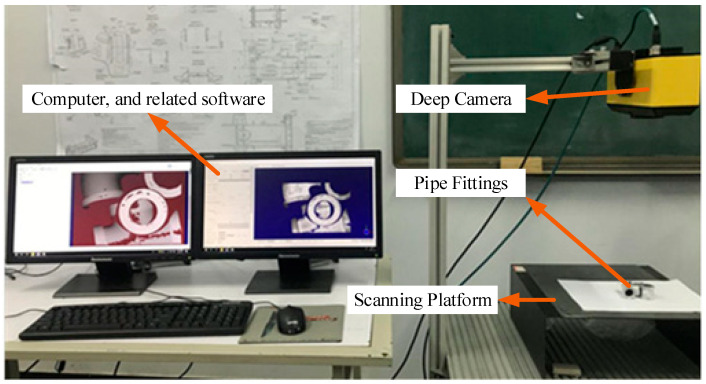
Experimental Equipment.

**Figure 12:**
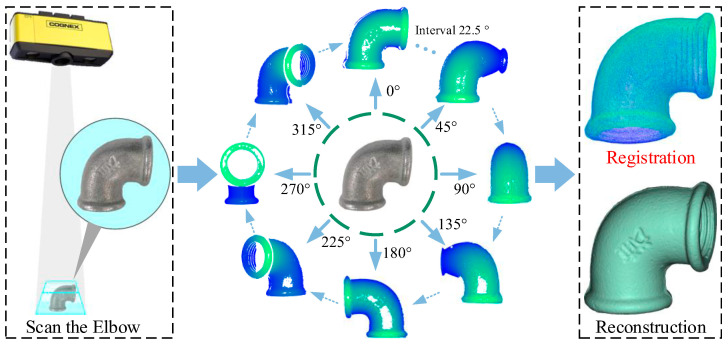
Multi-View Scanning and Registration.

**Figure 13:**
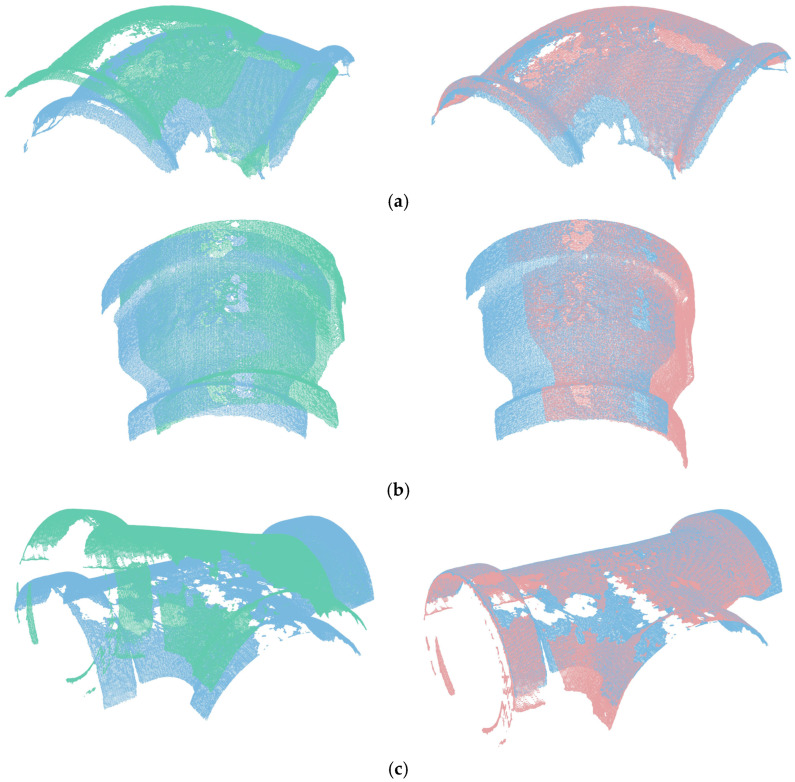
Registration Results of Point Clouds for Pipe Fittings (blue represents the target point cloud, green represents the source point cloud, and orange represents the aligned point cloud). (**a**) elbow01&02; (**b**) reducer01&02; (**c**) tee01&02.

The experimental data was sourced from an industrial pipe fitting point cloud dataset, which was autonomously collected. Common industrial pipe fittings and a portion of the point clouds involved in the experiment are shown in Figure 10. The experimental equipment is shown in Figure 11. Data acquisition was performed using the COGNEX 3D-A5005 depth camera (Cognex Corp., Natick, MA, USA) along with its accompanying hardware and software system, capturing the 3D point cloud data of the fittings through multi-view scanning. The schematic diagram of multi-view scanning and registration is shown in Figure 12. In the registration experiment design, an odd-even index grouping strategy was adopted, with the odd-indexed point cloud serving as the target point cloud and the even-indexed point cloud as the source. To ensure data quality, all point clouds underwent a standardized preprocessing workflow, including pass-through filtering and voxel filtering (1/4 downsampling). Considering the uncertainty of a single registration experiment, we conducted 100 trials to ensure the reliability of the results. The registration results are shown in Table 6 and Figure 13. Table 6 records the registration errors of each algorithm under the same experimental conditions, while Figure 13 displays the registration results of the proposed algorithm.

As shown in Figure 10c–e, the high reflectivity of the metallic surface of the pipe fittings leads to data loss in the point cloud captured by the depth camera. This data loss increases the complexity and difficulty of the point cloud registration process. Utilizing the DANIP algorithm, which is more robust to data loss, for coarse registration to provide better initial values effectively improves the registration accuracy.

As indicated by the experimental data in Table 6, the proposed coarse-to-fine point cloud registration algorithm based on DANIP and ICP demonstrates outstanding performance in terms of registration accuracy, with the root mean square error *RMSE* consistently lower than that of the comparison methods, yielding the best registration results. As seen in Figure 13, even in the presence of significant data loss in both the source and target point clouds, the coarse-to-fine registration algorithm based on DANIP and ICP can still achieve precise registration.

The experimental results confirm that the coarse-to-fine registration algorithm based on DANIP and ICP effectively overcomes the limitations of traditional methods when handling data loss caused by surface reflectivity. It significantly improves registration accuracy. This not only validates the robustness and reliability of the algorithm in real-world pipe fitting point cloud registration scenarios but also provides a new solution for future research and practical applications.

## 5. Conclusions and Future Work

This paper introduces a novel 3D point cloud keypoint detection method—Density-Aware Normal Inner Product Keypoint Detection (DANIP). DANIP incorporates a density-based edge point removal mechanism and utilizes adaptive neighborhood normal vector inner products for keypoint detection. The method demonstrates superior performance in terms of recall rate, precision, and computational efficiency. Furthermore, a coarse-to-fine point cloud registration method combining DANIP and ICP is proposed. This method leverages the robust performance of DANIP in handling data missingness for coarse registration, effectively addressing the issues of ICP’s susceptibility to local minima and low efficiency. The main research conclusions are as follows:

1. A novel keypoint detection method, DANIP, is proposed. Experimental results in keypoint detection show that, compared to other classical methods, DANIP achieves higher detection accuracy and computational efficiency on public datasets such as Stanford, Kinect, Queen, and ASL-LRD.
2. A coarse-to-fine registration method combining DANIP and ICP is proposed. This method effectively avoids the local minima problem in the ICP algorithm, significantly improving convergence efficiency and computational performance. Under optimal conditions, the runtime is reduced by 66.93%, 78.01%, 75.48%, and 23.69% on the Stanford, Kinect, Queen, and ASL-LRD datasets, respectively.
3. Compared to other classical registration algorithms, the coarse-to-fine point cloud registration based on DANIP and ICP achieves higher accuracy even in the presence of severe data loss in multi-view industrial pipe datasets. These findings validate the robustness of the proposed method against data loss caused by reflectivity and highlight its potential in engineering applications.

However, despite the promising results, several limitations persist. While the method performs well on standard datasets, its applicability to more complex or noisy real-world data requires further investigation. Additionally, although the method handles moderate data loss effectively, it still faces challenges when dealing with severe or systematic data loss.

In future research, we plan to further combine improvements in the algorithm with the effects of actual industrial applications, focusing on the following aspects: 

Production Efficiency Improvement: By integrating industrial field data, we will assess the impact of the registration algorithm on production efficiency. By optimizing the algorithm’s runtime, we aim to increase the number of parts produced per hour and compare this with traditional methods to analyze its potential industrial value.

Control of Geometric Tolerances and Precision Enhancement: We will conduct in-depth research on how the algorithm ensures the geometric tolerances of machined parts. Additionally, we will explore how precise registration control can achieve higher assembly accuracy and reduce assembly errors.

Reduction in Rework Rates and Quality Improvement: The improvement in algorithm accuracy will directly impact assembly precision, reducing rework rates caused by errors. Future studies will verify the practical effects of the algorithm in reducing rework rates and improving production quality through experimental data.

## Figures and Tables

**Table 1:** Datasets used in the evaluation.

No.	Datasets	Acquisition Method	Characteristic	Quality	Degree of Data Loss	Model Number
1	Stanford	Cyberware 3030 MS	Diversity	High	Low	6
2	Kinect	Microsoft Kinect	Low density	Low	Medium	7
3	Queen	Minolta vivid	Scanning error	Medium	Medium	5
4	ASL-LRD	Hokuyo UTM-30LX	Large size and noise	Medium	High	8

**Table 2:** *F*_1_ Score and Time of Keypoint Detection.

Parameter	Datasets	ISS	Harris 3D	NVDP	SIFT 3D	DANIP
*F*_1_	Stanford	0.9326	0.7415	0.8939	**0.9645**	0.9512
	Kinect	0.7729	0.8162	0.9162	**0.9443**	0.9302
	Queen	0.9821	0.9503	0.9240	1.0000	**1.0000**
	ASL-LRD	0.7468	0.8416	0.9771	0.9677	**0.9891**
*Time*(s)	Stanford	**0.7471**	1.0388	0.8571	2.8569	0.8879
	Kinect	0.2384	0.2201	**0.1676**	2.0467	0.3374
	Queen	0.2073	**0.0941**	0.1399	1.7302	0.2001
	ASL-LRD	0.4775	**0.3389**	0.5979	3.1702	1.0059

**Table 3:** Comparison of Parameters for Different Datasets and Methods with 100 Repetitions.

Parameter	Datasets	ISS	Harris 3D	SIFT 3D	SUSAN	NVDP	DANIP
*RMSE*	Stanford	0.0023	0.0025	0.0023	0.0042	0.0024	**0.0022**
	Kinect	0.0052	0.0059	**0.0045**	0.0065	0.0055	0.0047
	Queen	0.0191	0.0190	**0.0181**	0.0203	0.0197	**0.0181**
	ASL-LRD	0.2024	0.1780	0.1334	0.1812	0.1407	**0.1288**
*MRE*	Stanford	0.000947	0.001312	0.000896	0.002344	0.001065	**0.000855**
	Kinect	0.003975	0.004772	**0.002948**	0.005016	0.004028	0.003406
	Queen	0.011691	0.010521	0.009631	0.012468	0.012118	**0.009432**
	ASL-LRD	0.137210	0.112940	0.072324	0.113120	0.093307	**0.065782**
*BIC*	Stanford	71.1403	71.2232	71.1121	71.4602	71.1886	**71.0851**
	Kinect	64.2029	64.3675	**64.1186**	64.3946	64.3104	64.1285
	Queen	64.0053	64.0011	63.9651	64.1606	64.0278	**63.9607**
	ASL-LRD	79.9788	78.0622	76.4428	78.4243	76.6458	**76.2269**
*Time*(s)	Stanford	2.92	4.32	4.79	4.33	2.93	**2.44**
	Kinect	2.49	2.10	4.26	3.07	1.75	**1.26**
	Queen	1.44	2.35	4.10	2.24	2.09	**1.21**
	ASL-LRD	3.52	4.27	5.90	3.09	3.42	**3.32**

**Table 4:** RMSE Summary of Registration Errors.

Parameter	Datasets	ISS	Harris 3D	SIFT 3D	SUSAN	NVDP	DANIP
Medians	Stanford	0.00237	0.00251	0.00228	0.00441	0.00243	**0.00224**
	Kinect	0.00518	0.00587	**0.00453**	0.00663	0.00554	0.00472
	Queen	0.01917	0.01902	0.01816	0.02026	0.01975	**0.01798**
	ASL-LRD	0.19853	0.17973	0.14412	0.19213	0.14572	**0.12919**
IQR	Stanford	0.00008	0.00028	0.00012	0.00052	0.00007	**0.00003**
	Kinect	0.000212	0.000336	0.000164	0.000735	0.000107	0.000131
	Queen	0.001475	**0.000821**	0.000902	0.001015	0.001242	0.000901
	ASL-LRD	0.002019	0.001826	0.001263	0.001938	0.001312	**0.000553**

**Table 5:** Performance Comparison of Algorithms Across Different Datasets with 100 Repetitions.

Algorithm	Metric	Stanford bun000&045	Kinect PeterRabbit000&001	Queen im0&2	ASL-LRD Hokuyo 0&1
ICP	Runtime(s)	14.3966	12.6766	9.7429	7.1939
SUSAN + ICP	Runtime(s)	7.9865	5.2159	3.1650	6.1760
	Rate of decline	44.53%	58.85%	67.51%	14.15%
Harris 3D + ICP	Runtime(s)	7.3926	3.7220	3.6199	6.6802
	Rate of decline	48.65%	70.64%	62.85%	7.14%
NVDP + ICP	Runtime(s)	5.3924	3.1112	3.1004	5.7802
	Rate of decline	62.54%	75.46%	68.18%	19.65%
ISS + ICP	Runtime(s)	6.5340	4.0449	2.7233	6.0138
	Rate of decline	54.61%	68.09%	72.05%	16.40%
SIFT 3D + ICP	Runtime(s)	7.5215	5.7851	5.3045	8.3011
	Rate of decline	47.76%	54.36%	45.56%	N/A
DANIP + ICP	Runtime(s)	**4.7604**	**2.7873**	**2.3889**	**5.4896**
	Rate of decline	**66.93%**	**78.01%**	**75.48%**	**23.69%**

**Table 6:** The registration error *RMSE* of commonly used pipe fittings.

Point Cloud	Number of Point	ICP	LM-ICP	P-ICP	G-ICP	NDT	DANIP-ICP
elbow01&02	131,071&139,978	0.5011	0.4951	0.5085	0.5082	0.7039	**0.4935**
elbow03&04	127,826&122,986	0.3937	0.3793	0.4996	0.5201	1.2531	**0.3778**
elbow07&08	123,277&115,856	0.7988	0.7983	0.5057	0.5118	1.3206	**0.4327**
elbow15&16	121,585&122,655	0.7716	0.7719	0.8615	0.8948	2.3735	**0.7712**
reducer01&02	84,686&85,298	0.2289	0.2191	0.2409	0.3386	0.2278	**0.2151**
reducer03&04	83,436&89,881	0.4864	0.4890	0.6087	0.6603	0.4129	**0.4063**
tee01&02	153,909&149,825	1.3562	1.3533	1.3998	1.4392	2.8303	**1.3433**

## Data Availability

The raw data supporting the conclusions of this article will be made available by the authors on request.
